# An Evidence-Based Public Health Approach to Climate Change Adaptation

**DOI:** 10.1289/ehp.1307396

**Published:** 2014-07-08

**Authors:** Jeremy J. Hess, Millicent Eidson, Jennifer E. Tlumak, Kristin K. Raab, George Luber

**Affiliations:** 1 Climate and Health Program, Division of Environmental Hazards and Health Effects, National Center for Environmental Health, Centers for Disease Control and Prevention, Atlanta, Georgia, USA; 2 Department of Environmental Health, Rollins School of Public Health at Emory University, Atlanta, Georgia, USA; 3 Department of Emergency Medicine, Emory University School of Medicine, Emory University, Atlanta, Georgia, USA; 4 Office of Public Health Practice, New York State Department of Health, Albany, New York, USA; 5 Department of Epidemiology and Biostatistics, University of Albany School of Public Health, Albany, New York, USA; 6 Environmental Health Division, Minnesota Department of Health, St. Paul, Minnesota, USA

## Abstract

Background: Public health is committed to evidence-based practice, yet there has been minimal discussion of how to apply an evidence-based practice framework to climate change adaptation.

Objectives: Our goal was to review the literature on evidence-based public health (EBPH), to determine whether it can be applied to climate change adaptation, and to consider how emphasizing evidence-based practice may influence research and practice decisions related to public health adaptation to climate change.

Methods: We conducted a substantive review of EBPH, identified a consensus EBPH framework, and modified it to support an EBPH approach to climate change adaptation. We applied the framework to an example and considered implications for stakeholders.

Discussion: A modified EBPH framework can accommodate the wide range of exposures, outcomes, and modes of inquiry associated with climate change adaptation and the variety of settings in which adaptation activities will be pursued. Several factors currently limit application of the framework, including a lack of higher-level evidence of intervention efficacy and a lack of guidelines for reporting climate change health impact projections. To enhance the evidence base, there must be increased attention to designing, evaluating, and reporting adaptation interventions; standardized health impact projection reporting; and increased attention to knowledge translation. This approach has implications for funders, researchers, journal editors, practitioners, and policy makers.

Conclusions: The current approach to EBPH can, with modifications, support climate change adaptation activities, but there is little evidence regarding interventions and knowledge translation, and guidelines for projecting health impacts are lacking. Realizing the goal of an evidence-based approach will require systematic, coordinated efforts among various stakeholders.

Citation: Hess JJ, Eidson M, Tlumak JE, Raab KK, Luber G. 2014. An evidence-based public health approach to climate change adaptation. Environ Health Perspect 122:1177–1186; http://dx.doi.org/10.1289/ehp.1307396

## Background

For public health, climate change has gone from a possible concern to what appears to be an actual threat in just over two decades. When the health impacts of climate change were first broached in the health literature, they were referred to as a possible eventuality (Longstreth 1991). Climate change is now recognized by many as a significant public health threat with substantial current health burdens (Åström et al. 2013; McMichael et al. 2004) and additional impacts expected over time both domestically (Ebi et al. 2006b) and abroad (Campbell-Lendrum et al. 2007). Much work has gone into identifying the potential health impacts of climate change in various regions and into projecting health impacts, which range from respiratory and other diseases associated with worsening air quality (Fang et al. 2013) to injuries (Hambly et al. 2013) to nutrition (Jankowska et al. 2012), among others. Other work has gone into exploring the health impacts of climate change mitigation strategies, many of which appear salutary (Haines et al. 2010). As with any rapidly evolving issue, the fast transition from a theoretical possibility to an apparent threat has been a broad challenge, from risk assessment to preparedness planning (Schmidt 2009).

As climatic shifts have become increasingly dramatic in recent years (Hansen et al. 2012), public health and other sectors have turned their attention to climate change adaptation (CCA) (Bierbaum et al. 2013). At the same time, some have expressed concern that there is relatively little evidence to guide CCA efforts (Armstrong et al. 2012; Hosking and Campbell-Lendrum 2012). Behind this concern is a commitment to evidence-based practice, but as of yet there has been very little discussion regarding how the principles of evidence-based public health (EBPH) might be applied to CCA activities.

Whereas evidence has always been brought to bear in public health practice, the formal discipline of EBPH has evolved relatively recently and is now generally considered standard public health practice (Brownson et al. 2009). Here we review the concept of EBPH and outline a generally accepted framework as well as relevant controversies in the EBPH literature. Next, we consider how the EBPH framework might be applied to CCA and present a revised EBPH framework for CCA in public health. Finally, we consider implications of a commitment to EBPH adaptation to climate change for programming, research funding and reporting, and policy. Although we recognize the importance of climate change mitigation in reducing overall threats to public health and as a complement to adaptation efforts (Bierbaum et al. 2013), we confine our discussion here to adaptation efforts specifically.

## EBPH

*History and scope*. EBPH has been defined as “the development, implementation, and evaluation of effective programs and policies in public health through application of principles of scientific reasoning” (Baker et al. 2009). It is a process whereby evidence related to the nature and magnitude of public health problems and interventions to address them is systematically assembled, evaluated, and integrated into public health decision making. Its use is strongly supported, with few exceptions (Nadav and Dani 2006). Although there continues to be active debate regarding how to conceptualize and apply EBPH, its principles are esteemed and widely applied (Brownson et al. 2009, 2013).

As a concept, EBPH has evolved since it was introduced as a cousin of evidence-based medicine (EBM). EBPH theorists and practitioners have considered how evidence generation and application differ in the domain of public health compared with EBM. In response, EBPH has evolved to incorporate a framework with less emphasis on evidence hierarchy and more emphasis on knowledge translation—the process of synthesizing, disseminating, and applying knowledge to improve public health practice (Sudsawad 2007). As part of this evolution there has been widespread discussion of what EBPH should entail (Eriksson 2000), what dimensions of programming should be addressed (Glasgow et al. 1999), how different types of evidence should be evaluated (Rychetnik et al. 2002) and incorporated (Baker et al. 2008), guidelines for reporting different types of studies relevant to public health (Des Jarlais et al. 2004; Moher et al. 2009), implications for building the public health evidence base (Teutsch 2008), and efforts to collate and centralize evidence relevant to particular topics (Community Preventive Services Task Force 2012).

*Types of evidence used in EBPH and relevant reporting guidelines.* Review and evaluation of available evidence is a key requirement of EBPH (Rychetnik et al. 2004). Evidence is generally referred to as either qualitative or quantitative, depending on the methods used to generate it. Qualitative evidence relies on contextual data, which may be derived from upstream observation and analysis of community norms and behavior, with specific emphasis on particular populations, settings, and policy characteristics (Asthana and Halliday 2006; Baker et al. 2008, 2009). Quantitative evidence relies on empirical data and includes experimental methods—primarily randomized controlled trials (RCTs)—as well as quasi-experimental, time-series, and observational study designs (Brennan et al. 2011), which include ecological studies. Mathematical modeling efforts have also become increasingly common for generating quantitative evidence and occupy a distinct evidentiary niche. In the case of climate and health studies, specifically, such studies typically use projected outputs from global circulation models to generate modeled, spatially distributed exposure concentrations that are linked with known or modeled spatial population distributions (Huang et al. 2011).

Recent years have seen the development of reporting guidelines, standards, and critical appraisal checklists to guide reporting of scientific studies and help consumers evaluate the level and strength of evidence and other factors in systematic reviews (Rychetnik et al. 2004). Reporting guidelines are “evidence-based tools that employ expert consensus to specify minimum criteria for authors to report their research such that readers can both critically appraise and interpret study findings” (Bennett and Manuel 2012). Scientific journal editors and other groups are increasingly recommending use of some key guidelines and checklists to increase the clarity and transparency of scientific articles, with the ultimate hope of enhancing practitioners’ ability to evaluate the evidence. Many guidelines for reporting health research have been developed; for instance, a recent systematic review identified 81 sets of reporting guidelines for health research (Moher et al. 2011). Some of the key guidelines relevant to climate change and health, including those for RCTs, nonrandomized or observational studies, other or mixed-methods study types, meta-analyses and systematic reviews, and mathematical modeling, as well as brief descriptions and references for each tool, are summarized in Supplemental Material, Table S1. Also listed are examples of how the tools have been applied to specific public health concerns.

The Enhancing the Quality and Transparency of Health Research (EQUATOR) Network website (http://www.equator-network.org/) lists almost 200 health study reporting guidelines (Simera et al. 2012). An EQUATOR survey of 30 groups with guideline development processes found that only five (17%) had performed any formal evaluation of the impact of their guidelines on the quality of study reporting or uptake of the guidelines by journals (Simera et al. 2008). Scientists disagree on whether certain types of study design should inherently be respected as providing stronger evidence or whether study quality (i.e., adherence to consensus guidelines on study design, data analysis, and results reporting for particular methodological approaches) is more important. Although RCTs are often considered the “gold standard” for ideal study design because the randomization theoretically accounts for potential sources of bias, some researchers believe that RCTs can be deficient compared with dynamic modeling (Thompson 2010).

Even though modeling holds promise, there is great variation in approaches to modeling work and reporting of study results even in relatively narrowly defined applications such as the projection of climate change health impacts (Huang et al. 2011). Guidelines for mathematical modeling studies have only recently been developed, and only in regard to specific applications. Bennett and Manuel (2012) reviewed the literature for extant reporting guidelines on population modeling studies and found no specific guidelines, but did locate seven related studies and identified 69 quality criteria with distinct reporting characteristics. In part because the authors found little consistency among studies as to what to report, they suggested that population modeling studies could benefit from development of a specific reporting guideline (Bennett and Manuel 2012).

*Consensus regarding the process of EBPH*. In the rich, evolving EBPH literature, two major themes are apparent. First, there is general consensus regarding the process of EBPH. As summarized in a recent review, there are multiple core concepts central to an EBPH process: making decisions based on high-quality, peer-reviewed evidence; systematic use of data and information systems; application of program planning frameworks based in behavioral science theory; community engagement; program evaluation; transparent dissemination of evaluation findings; and synthesis of sound decision making and scientific skill in decision making (Brownson et al. 2013). In regard to applying evidence to interventions, specifically, a general consensus approach appears to have emerged and many tools are available to support its application (Latham et al. 2013). The general approach is widely referenced and taught (Jansen and Hoeijmakers 2013). The general approach is well exemplified in the six-step framework presented by Jones et al. (2010).

The first step in the EBPH process is “problem assessment,” in which public health concerns of interest are identified and surveillance and survey data are reviewed to evaluate associated disease burdens on a population basis. This stage necessarily involves characterization of the public health concern and associated outcomes, and often at least implicitly involves a logic model of the disease of interest, exposure pathways, and potential prevention strategies. This stage need not necessarily entail comparative risk assessment, although it follows from an evidence-based approach that consideration of relative disease burden should be a criterion for what problems are systematically addressed through EBPH. Several studies have demonstrated the range of problems to which EBPH has been applied, including injuries in the U.S. military (Jones et al. 2010), physical inactivity in Europe (Cavill et al. 2006), and low levels of health literacy among mothers (Levandowski et al. 2006).

The second step entails searching for and assembling evidence of effective prevention using multiple modalities—from systematic literature reviews (Jones et al. 2010) to focus groups (Cavill et al. 2006) to primary data analysis (Levandowski et al. 2006)—to assess the evidence for interventions that may reduce the incidence or burden of the public health impact. An example illustrates how this may be done on a national scale. England’s national public health agency commissioned a team of experts to produce evidence-based guidance on the effectiveness of physical activity interventions. Literature reviews and input from focus groups of “experienced physical activity practitioners” informed their product (Cavill et al. 2006). In addition, England’s 2004 Department of Health guidance, which provides a “comprehensive review of the global literature on the relationship between physical activity and health” (Cavill et al. 2006), became widely recognized as the document of choice for English investigators wanting a firm evidence base to justify action regarding physical activity (Cavill et al. 2006). In these ways, England’s national government coalesced evidence for effective physical activity interventions.

The third step of the framework entails evaluation of individual pieces of evidence assembled in the second step. In this stage, the reviewer “evaluates the quality of individual research studies using predetermined criteria to assess strengths and weaknesses of design, execution, and analysis” (Jones et al. 2010). These predetermined criteria are systematically applied to each of the studies identified in step two to determine whether the intervention(s) being considered is (are) effective and how consistent and strong the evidence in favor (or against) the interventions might be. As noted above, a wide range of different types of evidence is available, although the body of evidence on a particular topic may be more narrow. The framework described by Jones et al. (2010) is not prescriptive of a particular approach to assessing the evidence. The issue of what criteria should be used to evaluate the evidence is perhaps where there is the least consensus in the EBPH community, an issue to which we turn in greater depth below.

In the fourth step, the reviewer assesses the body of assembled evidence based on overall strength and consistency and translates the evidence into recommendations for public health interventions. Jones et al. (2010) noted that this step requires an inclusive approach: Multiple sources of evidence are required to adequately assess intervention effectiveness, potential harms, and implementation issues. Others have more recently further expanded the domain of this step to include consideration of various types of evidence related to various components of knowledge translation (Rychetnik et al. 2012).

Step 5 involves application of predetermined criteria to rank interventions based both on problem prioritization and evidence of intervention efficacy. This step integrates components of step one with step four to generate recommendations for specific interventions in a particular context. For example, Jones et al. (2010) identified different causes of fatal injury (mostly motor vehicle crashes) versus nonfatal injury (primarily falls and sports as well as motor vehicle accidents) within the military. These varying causes require varying approaches to prevention. Where significant evidence (including surveillance) exists and civilian models can be adopted, such as for motor vehicle crashes, interventions have been effective. In other cases, where little evidence for prevention within the military context exists, as with falls, prioritizing interventions may be hindered. Overall, Jones et al. (2010) argued that problems for which there exists a strong scientific evidence base should receive priority attention and that this evidence should be inclusive, containing fatality, disability, hospitalization, and outpatient data. Concerning intervention assessment, important considerations include characterizing associated health outcomes, the magnitude of effect, and assessing both positive and negative effects. Rather than recommending specific criteria for setting intervention priorities, Jones et al. (2010) discussed major categories: effectiveness, costs, feasibility, acceptability, and sustainability. In ranking the competing intervention options, quantitative and qualitative approaches can be used; Jones et al. (2010) highlighted a particular set of priority-setting criteria used by military working groups to address injury prevention.

The sixth and final step in the framework entails identification of limitations of the current research and remaining research gaps. For example, Levandowski et al. (2006) identified study limitations that included the use of maternal educational attainment as a proxy for maternal literacy, an inability to tease out the separate impact of health literacy training from the overall intervention impact, and the incomplete applicability of vital records data to the actual study population.

*Consensus regarding evidence hierarchies*. A second major theme in recent EBPH literature is that EBPH requires a different approach to evaluation of evidence than EBM, in that RCTs are placed at the top of the evidence hierarchy. Some EBPH researchers have questioned the prioritization of experimental evidence, noting that study quality may be more important than whether the study is experimental or observational (Petticrew and Roberts 2003; Rychetnik et al. 2002). In addition, they noted that the ideal research design is governed by the particular type of scientific question being addressed (Petticrew and Roberts 2003), and many public health questions cannot ethically or logistically be addressed with RCTs.

In general, EBPH practitioners have emphasized the public health need for a wider range of evidence types and suggest a less hierarchical approach to study methods (Brownson et al. 2009). Some have argued that, although experimental approaches are more likely to provide valid information regarding intervention efficacy, “study design alone is an inadequate marker of evidence quality in public health intervention evaluation” (Rychetnik et al. 2002) and that ranking experimental evidence more highly “will favor interventions with a medical rather than a social focus, those that target individuals rather than communities or populations, and those that focus on the influence of proximal rather than distal determinants of health” (Rychetnik et al. 2002).

Still others have rejected the emphasis on evidence hierarchies and asserted that evidence typologies should be used instead. Typologies can help conceptualize the strengths of various methodological approaches and match research questions to appropriate types of evidence (Petticrew and Roberts 2003). This perspective fits well with the emphasis on knowledge translation and acknowledgement of the wide range of settings in which public health evidence must be applied. Exploring these issues necessitates a wider range of research questions that speak more directly to the contextual specificity of the EBPH process in particular settings (Rychetnik et al. 2012). This also echoes practitioners’ commentary that highlights the lack of practice-relevant research (Green 2006; Hill et al. 2010) and laments the poor dissemination of relevant evidence, lack of in-house technology needed for evidence-based practice, and behavioral norms that do not support independent research (Kreuter and Bernhardt 2009).

In response to these concerns, there have been multiple efforts to develop systems that increase access to and the use of relevant evidence-based information (Dreisinger et al. 2008; Hill et al. 2010; Nieto et al. 2010; Twose et al. 2008). Most of these efforts have taken an inclusive approach to systematic review of the evidence. The *Guide to Community Preventive Services* (Community Preventive Services Task Force 2012) serves as an example. Consistent with this, recent scholarship on the topic has also focused on highlighting available tools for assessing data and the literature without making pronouncements regarding evidence quality and ranking (Jacobs et al. 2012).

*Summary*. In summary, it is clear that EBPH is now an important part of public health and that there is a generally accepted approach to the process. It is also clear that, although there is not complete consensus regarding evidence hierarchies, prioritizing experimental evidence may be important for interventions but is less so for implementation activities—that is, how interventions are carried out and what makes for successful implementation. Regardless, the consensus seems to hold that all forms of evidence bear consideration and evaluation and that evidence related to knowledge translation is particularly important for public health. With these considerations in mind, we can consider how the EBPH framework might be applied to the particular issues of CCA in public health.

## EBPH Adaptation to Climate Change

By its nature, EBPH tends to be applied to established health concerns with a significant evidence base, bounded impacts that are apparent historically and in present time, and for which administrative responses have been at least partially established. Because of the unique characteristics of climate change, applying EBPH to climate change and its health impacts poses special challenges.

Several issues arise in particular when considering how to apply EBPH to public health adaptation efforts. These issues can be grouped into two subsets: *a*) issues arising from the need to reconcile established approaches to EBPH and to CCA in public health, and *b*) issues arising from prioritizing an evidence-based approach. In the remainder of this section, we consider the first subset, specifically the questions of whether the established EBPH framework needs to be modified to accommodate established conventions of CCA in public health, how a modified framework might be applied, and whether such a framework would resonate with existing approaches. Following that discussion, we explore the second subset and examine how prioritizing an EBPH approach to CCA might affect programming, research, and policy making going forward.

*Modification of the established EBPH framework*. There is considerable literature on the process of CCA in public health, which entails a process of vulnerability assessment; identification of how relevant exposures and population vulnerabilities are likely to shift; and preparation, implementation, and evaluation of adaptation plans (Ebi and Burton 2008; Ebi and Semenza 2008; Ebi et al. 2005, 2006a). Much of this literature develops and elaborates frameworks for identifying climate-sensitive public health concerns, projecting and assessing future disease burdens based on comparative risk assessment using output from global circulation models coupled with exposure–outcome associations, and developing strategies to improve population resilience to adverse exposures. Relatively little of the literature is focused on specific interventions or on intervention implementation. It appears that, in large part, the activities identified as important components of CCA in public health—such as risk assessment, intervention development, and intervention evaluation—can be mapped relatively easily onto the established framework articulated by Jones et al. (2010), with some modifications. Table 1 presents both the framework of Jones et al. (2010) (in the first column) and a proposed modification (in subsequent columns) to accommodate the process of CCA in public health. The basic framework remains intact, and the modifications are primarily in the steps related to problem assessment.

**Table 1:** EBPH steps applied to climate change issue: extreme heat events.

EBPH step	Public health step	Health department activity	Measures used	Evidence used	Relevant guidelines
Step 1a. Problem assessment: definition of decision space	Identification of responsible party(ies): “State Health Department” identified for this example	Characterization and maintenance of relevant administrative pathways
Step 1b. Problem assessment: identification of biggest or most severe problems/assessment of the size of the problem through an integrated assessment or impact assessment	Hazard characterization	EHEs identified and defined	Magnitude, duration, return period	Weather and climate; general circulation model projections	None
	Risk characterization	EHE of particular magnitude and duration	Event rates, risk ratios, incidence rate ratios, odds ratios, hazard ratios; time-to-event (binary or continuous), DALYs, QALYs	Cross-sectional studies, cohort studies, case–control studies, systematic reviews, meta-analyses	CCRBT; PRISMA; ISPOR
	Risk projection	Projection of deaths from EHEs in a particular location at a particular time	Same as for risk characterization	Same as for risk characterization	CCRBT; PRISMA; ISPOR
	Assessment of current and anticipated public health capacity	EHE risk and preparedness for a given city/region over a given time frame	Multiple health outcomes and measures; scenario-based exercises	Multiple study types; performance in exercises; process and outcome measures	None
Step 2. Search for evidence of effective prevention/intervention efficacy	Systematic review/ literature review of prevention strategies	Literature review of EWS, EHE preparedness plans, and primary, secondary, and tertiary prevention strategies	Multiple health outcomes and measures	Multiple study types; systematic reviews; meta-analyses	Guide to Community Preventive Services methods; GRADE
Step 3. Evaluation/assessment of quality of evidence for prevention; identification of research gaps	Evaluate level of evidence for several potential interventions	Evaluate potential interventions: EWS, EHE preparedness plans, cooling centers, provision of air conditioners	Multiple health outcomes and measures	Evidence hierarchies; methodological appropriateness; typology of evidence	Guide to Community Preventive Services methods
Step 4. Recommendations based of strength and consistency of evidence	Recommend most promising interventions based on strength and consistency of evidence	Recommend interventions with the strongest level of evidence: EWS, EHE preparedness plans	Absolute or relative risk reduction, odds ratios, hazard ratios, cost estimates (e.g., value of a statistical life), DALYs, QALYs	Ecological studies, cohort studies, case–control studies, RCTs, systematic reviews, meta-analyses	Guide to Community Preventive Services methods; PRISMA; GRADE
Step 5. Prioritization of interventions	Use predetermined criteria to rank interventions; adaptation planning	Prioritization of interventions and determine intervention for implementation: implement EHE preparedness plan	Predetermined criteria such as effectiveness, cost, feasibility, acceptability, sustainability
Step 6. Intervention implementation and evaluation	Intervention implementation and process and impact evaluation	Evaluation of effectiveness of EHE preparedness plan in terms of process and impacts	Similar to step 4; indicators of process and impact evaluations	Similar to step 4; process and impact evaluations	ACE
Step 7. Identification of knowledge gaps/next steps	Identification of knowledge gaps and next steps	Information from all prior steps is used	All measures are used	All evidence is used
Abbreviations: ACE, Assessing Cost Effectiveness; CCRBT, Consolidated Standards of Reporting Trials; DALY, disability-adjusted life year; EHE, extreme heat event; EWS, early warning systems; GRADE, Grading of Recommendations Assessment, Development and Evaluation; ISPOR, International Society for Pharmacoeconomics and Outcomes Research; PRISMA, Preferred Reporting Items for Systematic Reviews and Meta-Analyses; RCT, randomized controlled trials; QALY, quality-adjusted life year. Adapted from Jones et al. (2010) with permission from the *American Journal of Preventive Medicine,* Elsevier. All rights reserved.					

The first modification includes division of the problem assessment step from the framework of Jones et al. (2010) into two components. This allows for the scoping and scaling activities used to identify the geographic and temporal scope of public health CCA efforts. For instance, step 1a—definition of the decision space—makes explicit the scoping consideration that adaptation activities are typically organized at a particular jurisdictional level [city or county, state (in the case of the United States), regional consortium, or country], and that CCA activities are often nested within multiple relevant jurisdictions. The second modification evident in the hazard characterization and risk characterization activities of step 1b allows for the explicit definition of the hazard being considered, (e.g., extreme heat events) and for adoption of a framework for characterizing public health risks associated with that hazard, for example, comparative risk assessment, one recommended approach to problem assessment for CCA activities (Campbell-Lendrum and Woodruff 2006), or health impact assessment (Patz et al. 2008). The third modification, also in step 1b, accommodates the use of projected harms, a fundamental component of climate and health preparedness, as well as ongoing surveillance data, the more common approach to prioritizing extant public health concerns. A fourth modification, again in step 1b, lies in explicit consideration of public health capacity at the same jurisdiction and time. Collectively, these modifications in step 1b allow for the requisite assembly of evidence of projected harms and consideration of a host of associated methodological concerns related to institutional adaptive capacity (e.g., Gosling et al. 2012; Huang et al. 2011). Finally, a fifth modification allows for the explicit addition of intervention monitoring and evaluation as a distinct step to satisfy the need for iterative management processes that have become a staple of CCA activities. For ease of presentation, we have also separated out step 6 of Jones et al. (2010), which has two parts, into two separate steps: steps 6 and 7 in Table 1.

*Applying the revised framework*. An example is used to clarify how the modified EBPH framework might be applied. Table 2 highlights relevant evidence for each step of the process for the concern of extreme heat in New York City, New York, which we chose on the basis of the availability of resources for the initial steps of an exploratory process for a separate systematic review modeled on the *Guide to Community Preventive Services* (Community Preventive Services Task Force 2012). Keyword/phrase searches were conducted using PubMed (http://www.ncbi.nlm.nih.gov/pubmed/), Google Scholar (http://scholar.google.com/), and Google (http://google.com/) for the following terms: “heat wave,” “heat event,” “extreme heat,” “extreme temperature,” “temperature event,” “intervention,” “adaptation,” “mitigation,” “warning,” “advisory,” “cooling center,” and “cool center.” Studies were used for Table 2 if they provided some locally relevant evidence—that is, evidence likely to apply to the populations in that region in regard to risk profiles, community and agency adaptive capacity, and historical and future climate. Study strengths and weaknesses were summarized, and studies were chosen to maximize the Community Guide criteria for suitability (greatest, moderate, or least) based on internal validity, and quality (good, fair, or limited) based on descriptions of the study population and interventions, sampling, measurement of exposures and outcomes, data analysis, interpretation of results, and other threats to validity. When available, studies chosen as examples of the steps in Table 2 focused on New York or neighboring states and Canadian provinces, to provide more focused evidence.

**Table 2:** Example of modified EBPH framework applied to extreme heat, using New York regionally relevant studies when available.

EBPH step	Public health step	Health department activity	Examples
Step 1a. Problem assessment: definition of decision space	Identification of responsible party(ies)	Characterization and maintenance of relevant administrative pathways	Identification of lead agency or department with primary administrative responsibility, partners and affiliated organizations, and need for collaboration with other sectors is important based on review of municipal heat wave response plans including NY City (Bernard and McGeehin 2004).
Step 1b. Problem assessment: identification and assessment of problem severity through an integrated or impact assessment	Hazard characterization	EHEs identified and defined	*a*) Retrospective evaluation of heat index and other indices in forecasting increased mortality risk in heat waves for NY City (Metzger et al. 2010); *b*) characterization of historical climatic trends in the Northeast and projections of climatic shifts over the 21st century for the region (Kunkel et al. 2013); *c*) novel climate change temperature projection changes in temperature mean and variability for NY and other cities demonstrates higher morbidity with change in both variables versus change in mean temperature alone (Gosling et al. 2009).
	Risk characterization	EHEs of particular magnitude and duration; associated exposure pathways and population health risks	*a*) Each degree above the threshold of the temperature–health effect curve in NY City was associated with increases in same-day hospitalizations for respiratory disease and lagged hospitalizations for cardiovascular disease (Lin et al. 2009); *b*) mortality increased for accidental and nonaccidental deaths throughout the month of heat-associated NY City blackout (Anderson and Bell 2012); *c*) during a heat-associated blackout in NY City, respiratory hospital admissions increased significantly (Lin et al. 2011); *d*) an increase in temperature during embryogenesis was associated with congenital cataracts in NY (Van Zutphen et al. 2012).
	Risk projection	Projection of deaths from EHEs in a particular location at a particular time	*a*) By midcentury, heat-related premature mortality is expected to increase in NY City region (Knowlton et al. 2007); *b*) excess respiratory admissions for NY due to excessive heat are projected to be 2–6 times higher in 2080–2099 than in 1991–2004 (Lin et al. 2012); *c*) climate change could cause increase in summer ozone-related asthma emergency department visits for children in NY City (Sheffield et al. 2011).
	Assessment of current and anticipated public health capacity	EHE risk and preparedness for a given city/region over a given time frame	*a*) Vulnerable NY subpopulations for acute renal failure hospitalization were identified (Fletcher et al. 2012); *b*) the heat vulnerability index was associated with respiratory hospitalizations in Massachusetts (Reid et al. 2012); *c*) field test of web application surveillance system assessed during Quebec heat wave (Toutant et al. 2011); *d*) interviews in four cities, including NY City, assessed barriers and strengths in heat health warning systems (Sampson et al. 2013).
Step 2. Search for evidence of effective prevention/intervention efficacy	Systematic review/ literature review of prevention strategies	Literature review of EWS, EHE preparedness plans, and primary, secondary, and tertiary prevention strategies	*a*) Systematic review for interventions to prevent heat-related adverse health outcomes (Bassil and Cole 2010); *b*) literature review of federal, tribal, state, and local adaptations, including barriers and best practices (Bierbaum et al. 2013); *c*) replicated search by public for fan use, acclimatization, medication, hydration, activity reduction, and air conditioner use (O’Connor et al. 2008).
Step 3. Evaluation/ assessment of quality of evidence for prevention and identification of research gaps	Evaluate level of evidence for several potential interventions	Evaluate potential interventions: EWS, EHE preparedness plans, cooling centers, provision of air conditioners	*a*) Cochrane systematic review of studies of fan use to prevent adverse health impacts in heat waves; no studies met quality standards for inclusion; existing studies yield conflicting results. Recommended high quality research to shed light on issue (Gupta et al. 2012); *b*) Montreal public education campaign evaluated on message penetration and likelihood for adoption of protection measures (Gosselin et al. 2010); *c*) public knowledge of heat events was evaluated in eastern and western Canadian cities without heat alert response systems (Alberini et al. 2011).
Step 4. Recommendations based of strength and consistency of evidence	Recommend most promising interventions based on evidence strength and consistency	Recommend interventions with the strongest level of evidence: EWS, EHE preparedness plans	*a*) Recommendations for buddy system and risk communication after 2011 NY City heat wave (Lane et al. 2013); *b*) Montreal’s heat response plan updated after 2010 heat wave based on data analysis (Price et al. 2013); *c*) Vancouver compared scenarios and triggers to develop simple approach for local use (Henderson and Kosatsky 2012).
Step 5. Prioritization of interventions	Use predetermined criteria to rank interventions; adaptation planning	Prioritization of interventions and determine intervention for implementation: implement EHE preparedness plan	*a*) Review of cost effectiveness of various strategies in Maryland for preventing heat exposure and managing heat related illness (Huang et al. 2013); *b*) Philadelphia warning system prioritized messaging, increased staffing and air conditioning, with excellent cost/benefit (Ebi et al. 2004); *c*) prioritized capacity development, engagement with relevant sectors, and continual evaluation of decision making processes (Bowen et al. 2014).
Step 6. Intervention implementation and evaluation	Intervention implementation and process and impact evaluation	Evaluation of effectiveness of EHE preparedness plan in terms of process and impacts	*a*) Implementation: Reviews heat wave response plan contents, strengths, weaknesses for cities including NY (Bernard and McGeehin 2004); *b*) Evaluation: Ecological evaluation of heat response systems based on observed versus predicted excess mortality in France from 2003 to 2006; 2006 mortality lower than expected; concludes that heat warning system and response plan was effective (Fouillet et al. 2008); *c*) Evaluation: Comprehensive review mentioned above catalogs methods used to evaluate interventions to prevent heat-related adverse health outcomes and concludes that interventions are likely protective but notes lack of evidence of efficacy with most vulnerable populations; suggests developing a framework for evaluating public health interventions for heat and further studies on vulnerable populations (Bassil and Cole 2010).
Step 7. Identification of knowledge gaps/next steps	Identification of gaps in knowledge and next steps	Use information from steps 1–6 to identify knowledge gaps in problem assessment, prevention, and evaluation and to determine next steps	*a*) Systematic review cited above; found no high-quality evidence and recommended high quality research to shed light on issue (Gupta et al. 2012); *b*) Systematic review cited above highlights evidence gaps and suggests further evaluative studies on vulnerable populations and developing a framework for evaluating public health interventions for heat (Bassil and Cole 2010).
Abbreviations: EHE, extreme heat event; EWS, early warning systems; NY, New York.			

The example highlights several important dimensions of risks related to extreme heat in the region and also significant knowledge gaps. The surveyed literature revealed that there are several dimensions to the hazard in question, including extreme heat event magnitude, duration, and the role of temperature variability in population vulnerability. It also highlighted the diversity of health risks associated with this one relatively narrowly defined exposure. Evident also was the potential for cascading or compound risks, such as blackouts during extreme heat events, and that these events have their own distinct risk profiles.

Other insights from the application of the framework relate to limitations in the evidence base, particularly affecting steps 2–5. First, evidence is not centrally located currently, and many studies have not been systematically evaluated or assessed via an established review mechanism such as a Cochrane review (http://summaries.cochrane.org/) or the *Guide to Community Preventive Services* (Community Preventive Services Task Force 2012). Parties interested in using an EBPH approach for preventing and managing climate-sensitive diseases currently have few ready-made resources at their disposal on which to build.

Second, the volume and specificity of the evidence decreases progressively: There is substantial evidence to review regarding hazard characterization in step 1b (the evidence presented is a relatively small sample of what is available regarding climate projections for the region), but there is considerably less regionally specific information regarding intervention implementation and efficacy [both generally and specific to the identified decision space (step 2)], which affects successive steps in the framework (steps 4–6).

Third, although Table 1 focuses on published evidence, several cells in the table could also be filled through unpublished analyses of locally available data. For instance, a health department might review the efficacy of its local heat early warnings similar to evaluations done in other jurisdictions (e.g., Ebi et al. 2004) but not publish the findings. Such evidence would be highly relevant for local decision making. In addition, even if a jurisdiction has reviewed evidence as part of steps 3, 4, and 5, its review and assessment might not be published in the peer-reviewed literature given resource constraints and the priority of programming over publication.

Fourth, and perhaps most important, there is a dearth of relevant evidence overall, particularly in the areas of intervention efficacy and implementation. We chose the example of extreme heat in New York given the resources and studies available for such a review in that area. Nevertheless, despite a high level of climate and health expertise in New York’s academic and public sectors and a substantial commitment on the part of the regional governments to characterize and prepare for risks associated with climate change, evidence that can be used to select specific interventions for extreme heat and to guide their implementation is relatively rare. A recent systematic review of evidence related to other climate-sensitive diseases found little evidence related to interventions to reduce impacts for other climate-sensitive conditions, as well (Bouzid et al. 2013). Thus, an implication of the application of this framework is that rigorously evaluating the efficacy of candidate interventions should be a priority.

The extreme heat example is meant to be illustrative, but it is important to note that applying the framework to a different climate-sensitive health concern, for example, air pollution, would likely yield a very different set of findings. There would be more (or less) evidence in each category, different levels of agreement in the identified evidence for both problem assessment and adaptation interventions, and different intervention priorities. In some cases, for example, vector-borne and zoonotic diseases, potential harms from interventions will need to be considered alongside potential benefits and appropriate risk–benefit decisions made. Space considerations preclude exploration of other examples here.

*Integrating the revised framework into other existing approaches*. Although the above-mentioned review by Bouzid et al. (2013) and two other systematic reviews (Gupta et al. 2012; Stanke et al. 2013) represent recent applications of EBPH to climate-sensitive conditions, the framework has not been widely applied to specific climate and health topics. More commonly, evidence has been assembled through assessments such as those organized by the Intergovernmental Panel on Climate Change, which tend to focus primarily on problem assessment and less, in the case of public health, on evidence related to effectiveness of interventions to reduce impacts.

In addition to such assessments, there are multiple published vulnerability assessments that use a common methodological approach advanced by Ebi et al. (2006a). Most of the concepts advanced here for adapting the existing EBPH framework to CCA are touched upon in the Ebi framework, and the two frameworks are generally complementary: Vulnerability assessments highlight current capacity and ways in which future changes in climate may stress systems already in place (whereas this is a component of EBPH, it is less emphasized than in the vulnerability assessment framework), and EBPH is useful for prioritizing interventions based on efficacy. Two potentially significant differences are worth noting, however. First, vulnerability assessments focus significantly on problem assessment to capture both current and future impacts on the health sector as well as health impacts in other sectors. Impacts in other sectors [for example, shifts in agricultural productivity, changes in energy production and impacts on transportation infrastructure (Schneider et al. 2007)] are important to include given that they may affect public health indirectly. Second, vulnerability assessments are less explicit than EBPH reviews in their stipulation of criteria for evaluating evidence strength and consistency in comparison with major current EBPH efforts that place a strong emphasis on review methods [e.g., the *Guide to Community Preventive Services* (Community Preventive Services Task Force 2012)].

Despite these differences, the vulnerability assessment and the EBPH frameworks ultimately have much in common. The vulnerability assessment framework’s emphasis on impacts in other sectors highlights an important source of public health risk that the EBPH framework might otherwise miss if not specifically scoped and scaled to include these considerations in step 1. The EBPH framework introduces a formal process of evidence evaluation (steps 2–5) that will be increasingly important as adaptation activities come online and the need for evidence of effective adaptation policies becomes more important (Fankhauser et al. 1999; Lempert and Schlesinger 2001).

## Implications of Taking an Evidence-Based Approach

Regardless of the underlying framework used, committing to an evidence-based approach to CCA in public health has several potential implications and is likely to pose several significant challenges, as other authors have noted (e.g., Bouzid et al. 2013). Some of the most significant are better adherence to guidelines in climate and health research by both researchers and journal editors; enhanced attention to indicators that can be used in surveillance and research across various settings; greater attention by researchers and practitioners to study design and reporting for studies evaluating interventions, including consideration of when more robust experimental or quasi-experimental methods may be required; development of new reporting guidelines for modeling studies in climate and health research; explicit consideration of knowledge translation concerns specific to CCA; and better methods to inform and educate funders, policy makers, and practitioners on the benefits of and specific actions that advance an evidence-based approach to CCA. Below we expand on each implication in turn.

*Guideline adherence*. Consensus reporting guidelines, particularly if incorporated into editorial standards, should translate into better reporting of study design elements and findings, thereby enhancing the quality of studies adhering to reporting guidelines as well as study intercomparability and allowing for systematic review using Grading of Recommendations Assessment, Development and Evaluation (GRADE) scoring methods (Bouzid et al. 2013). Although evidence of guideline impact on study quality and incomparability is still pending, three changes are worth promoting: Consumers of scientific evidence—including those conducting assessments of the literature for policymakers—related to CCA in public health can employ consensus guidelines when assessing evidence; authors can familiarize themselves with relevant guidelines and include consensus elements in manuscripts; and editors of scientific journals publishing research relevant to CCA in public health can urge authors to adhere to established reporting guidelines. These three overlapping efforts have the potential to significantly improve the usability of evidence related to climate and health without limiting the emergence of new evidence in this young field.

*Indicators*. Indicators are “quantitative summary measures that can be used to track changes in conditions by person, place, and time” (English et al. 2009). Consensus regarding indicators of vulnerability to and preparedness for climate change is key for systematic quantification and tracking of both preparedness and climate-sensitive health outcomes over time (English et al. 2009). The effort to develop consensus indicators is still in process. Several organizations, including the World Health Organization–Europe, the U.S. Environmental Protection Agency (EPA), the National Research Council, the California EPA, and the State Environmental Health Indicator Collaborative (SEHIC), a workgroup of the Council of State and Territorial Epidemiologists (CSTE), have identified potential indicators, which English et al. (2009) reviewed and assessed for readiness in practice. Although proposed indicators cover a range of impacts and preparedness (e.g., excess morbidity and mortality associated with heat, counts of injuries and mortality associated with extreme weather events, numbers of heat early-warning systems, numbers of surveillance systems designed to capture climate change health impacts), the list is incomplete, and not all are equally ready for implementation in surveillance and tracking. Further work to develop robust indicators is ongoing as part of the National Climate Assessment (U.S. Global Change Research Program 2013) and will need to be cross-referenced with ongoing environmental public health tracking activity at the state and national levels.

*Study design and reporting*. Table 1 highlights the wide range of evidence types present in the climate and health literature, including observational, experimental, and modeling studies, systematic reviews, and meta-analyses. Most currently available evidence is observational, although modeling (i.e., projections of climate change health impacts) is relied on heavily in problem assessment. Experimental evidence is used rarely if at all, depending on the outcome of interest. Based on the current state of the evidence and existing guidelines for reporting, we can draw two conclusions: First, future research should aim to develop evidence typologies specific to climate and health (e.g., problem assessment, intervention evaluation, and modeling of projected impacts); second, appropriate methods including experimental and quasi-experimental designs should be explored in order to develop the evidence base regarding interventions relevant to CCA so practitioners can have higher confidence in their adaptation choices. Developing methods that could build the evidence base in lower-resource settings such as developing countries, where many impacts are expected, would be a particular boon.

*Guidance for modeling projected climate change health impacts*. The problem assessment step of the modified EBPH framework relies heavily on modeled projections of climate change health impacts based on scenario-based global circulation model outputs linked with concentration–response (exposure–outcome) functions. Researchers projecting climate change health impacts have taken a wide variety of approaches, some dynamic, some equilibrium, and some mechanistic, and as a result, approaches to model construction, sensitivity testing of results, and reporting vary. Most work to date has focused on various scenarios of heat exposure (Huang et al. 2011), although there are examples of projections of multiple other impacts, including respiratory disease (Orru et al. 2013), infectious diseases (Moors et al. 2013; Ogden et al. 2014), and malnutrition (Lloyd et al. 2011). The work of projecting climate change health impacts is fundamental to EBPH because it facilitates problem assessment. However, there is currently no consensus regarding required elements in the design and reporting of these studies. For instance, in a recent review of heat health impact projections, Huang et al. (2011) found a wide variety of methods and reporting conventions that precluded meta-analysis. There is therefore a need for discussion of these issues and the potential for developing consensus guidelines around the reporting requirements for climate health-impact projections [perhaps based on guidelines for other types of modeling (e.g., Weinstein et al. 2003)] to facilitate intercomparison of projected impacts for problem assessment. Such an effort might be organized by researchers and journal editors independently [as was the case with Consolidated Standards of Reporting Trials (CONSORT; http://www.consort-statement.org/)] or sponsored by a leading public health professional association or funder.

*Knowledge translation guidance*. The literature on knowledge translation is growing alongside the literature on climate and health. As the need for CCA becomes more apparent and a greater number of adaptation decisions affecting public health are made, it will be important to capture the rationale for, context of, and results of these decisions in the literature. As Rychetnik et al. (2012) noted, there are several relevant activities and research domains. Relevant activities include the review of evidence transferability (evaluation of existing evidence to determine whether interventions are likely to succeed in other settings) and knowledge translation (adoption of policies and strategies to promote uptake of evidence-based approaches). Relevant research includes translation research (empirical research applying interventions proven in one context to others and evaluating factors affecting applicability), and knowledge translation research [empirical research into development and testing of knowledge translation strategies (Rychetnik et al. 2012)]. As Rychetnik et al. (2012) and others have noted, there are several challenges in the field, including the need for frameworks to assess transferability (Milat et al. 2012) and strategies for generating comparable observations of programs across settings, measurement of intervention uptake, and strategies for scaling up interventions to the population level (Rychetnik et al. 2012). All of these concerns apply to climate and health programming generally and in specific areas such as disaster preparedness.

*Coordinated attention to adopting an evidence-based approach*. Climate change is a significant and looming public health threat. Whereas evidence related to projected health impacts is mounting, evidence related to adaptation interventions is lacking. A recent systematic review of evidence of intervention efficacy related to several climate-sensitive conditions found significant gaps in primary evidence, particularly for areas such as extreme weather events, droughts, floods, air pollution, and food security, as well as variability in prior assessments of the evidence that is available (Bouzid et al. 2013). Those who fund, generate, disseminate, and apply evidence to CCA can each take various actions to support the adoption of an evidence-based approach to CCA. Funders interested in endorsing an EBPH approach may want to increase their capacity to identify well-designed studies that address urgent population health needs and employ experimental and quasi-experimental methods to assess intervention impact. Funders may also consider allocating resources to knowledge translation as well as knowledge generation so that scientific advances are quickly translated into practice settings. To build the evidence base so that it supports intervention activity, researchers may consider prioritizing problems with real-world applications, sometimes in close collaboration with practitioners. They may also consider how CCA will likely require the study of interventions that are more local and specific to certain high-risk groups, in contrast to major environmental health issues such as air pollution in which evidence has focused on harm in order to substantiate large-scale engineering and policy solutions. To facilitate the dissemination of evidence, journal editors may want to consider how to prioritize studies of adaptation interventions alongside studies establishing harm and to require adherence to established reporting guidelines. They can also support development of guidelines in areas where there are none, for example, climate change health-impact projection studies. To promote the development of a substantial, high-quality evidence base, practitioners can continue to emphasize the need for intervention and knowledge translation research relevant to CCA and to use rigorous methods to assess, apply, and translate evidence in their public health practice. Finally, policy makers can use evidence-based policies to highlight the importance of a strong evidence base and support investments in expanding the evidence of intervention efficacy and knowledge translation.

## Conclusion

The need for CCA has become apparent, yet there is little evidence to guide adaptation decisions. EBPH has emerged as a powerful framework for assessing public health concerns and identifying the most effective health protection strategies. With some modifications, the existing EBPH framework can be applied to public health adaptation to climate change, supplementing existing approaches to making adaptation decisions. Adoption of an evidence-based approach presents several challenges for the field, and funders, researchers, journal editors, practitioners, and policy makers can each consider how their single and combined actions can lead to the realization of the potential of an evidence-based approach to public health adaptation to climate change.

## Supplemental Material

(266 KB) PDF
