# Supplementary material for: An Evidence-Based Public Health Approach to Climate Change Adaptation
Source: Environ Health Perspect. 2014 Jul 8;122(11):1177–86. doi: 10.1289/ehp.1307396 (PMC4216160; doi:10.1289/ehp.1307396)
Supplement: (266 KB) PDF [file ehp.1307396.s001.508.pdf]

Environmental Health Perspectives doi:10.1289/ehp.1307396

Supplemental Material

An Evidence-Based Public Health Approach to Climate Change Adaptation

Jeremy J. Hess, Millicent Eidson, Jennifer E. Tlumak, Kristin K. Raab, and George Luber

Table S1. Evidence-based public health tools with potential relevance to climate change.

Tools	Description	Examples
Consolidated Standards of Reporting Trials (CONSORT) (Moher et al. 2010; Schulz et al. 2010)	Checklist, flow diagram to improve transparency of randomized clinical trial reporting; Does not clearly evaluate strength of evidence	Respiratory research (Lu et al. 2013)
Cochrane Collaboration Risk of Bias (CCRBT) (Armijo-Olivo et al. 2012)	Grades on bias risk; Inter-rater reliability of grade--fair/ moderate; Inter-rater reliability across domains--slight	Electric fans and heatwaves (Gupta et al. 2012)
Strengthening the Reporting of Observational Studies in Epidemiology (STROBE) (Vandenbroucke et al. 2007; von Elm et al. 2008)	Checklists for evaluating and reporting information; Not designed to evaluate strength of evidence for study conclusions	Strategy to reduce pertussis (Wiley et al. 2013)
Value of Information (Petticrew et al. 2012)	Flow chart to assess strength and type of public health evidence	Motorcycle health impact (Petticrew et al. 2012)
Transparent Reporting of Evaluations with Non-randomized Designs (TREND) (Des Jarlais et al. 2004)	CONSORT type checklist to improve quality of data reporting in intervention evaluation studies with nonrandomized designs	Evaluating educational curriculum (Schulz et al. 2013)
Effective Public Health Practice Project Quality Assessment Tool (EPHPP) (Armijo-Olivo et al. 2012)	Characterize studies as weak, moderate or strong; Inter-rater reliability is strong; Poor agreement with CCRBT; Training essential to increase reliability	Environmental health risk communication (Fitzpatrick-Lewis et al. 2010)
Grading of Recommendations Assessment, Development and Evaluation (GRADE) (Guyatt et al. 2008)	Grades quality of evidence and strength of recommendation	Climate change and health interventions (Bouzid et al. 2013)
Guide to Community Prevention Services-Community Guide (Briss et al. 2004)	Evaluate effectiveness, study execution, design suitability, effect size, and other criteria	Asthma intervention (Crocker et al. 2011)
Assessing Cost Effectiveness (ACE) (Carter et al. 2009)	Evaluate multiple prevention program studies	Obesity (Carter et al. 2009)
Preferred Reporting Items for Systematic Reviews and Meta-Analyses (PRISMA) (Moher et al. 2009)	Designed for randomized clinical trials but suggested for other study types	Quality of climate change research (Hosking and Campbell-Lendrum 2012)
Meta-Analysis of Observational Studies in Epidemiology (MOOSE) (Stroup et al. 2000)	Checklist for sections of research papers	Asthma and obesity (Papoutsakis et al. 2013)
Institute of Medicine standards (Eden et al. 2011)	Assessing studies, synthesizing body of evidence, reporting	Clinical practice guidelines (Kung et al. 2012)
International Society for Pharmacoeconomics and Outcomes Research (ISPOR) Task Force on Good Research Practices—Modeling (Weinstein et al. 2003)	Assess model structure and data, validate	Microsimulation of health care costs (Glied and Tilipman 2010)

References

Armijo-Olivo S, Stiles CR, Hagen NA, Biondo PD, Cummings GG. 2012. Assessment of study quality for systematic reviews: a comparison of the Cochrane Collaboration Risk of Bias Tool and the Effective Public Health Practice Project Quality Assessment Tool: methodological research. J Eval Clin Pract 18(1):12-18.

Briss PA, Brownson RC, Fielding JE, Zaza S. 2004. Developing and using the Guide to Community Preventive Services: lessons learned about evidence-based public health. Annu Rev Public Health 25:281-302.

Carter R, Moodie M, Markwick A, Magnus A, Vos T, Swinburn B, et al. 2009. Assessing cost-effectiveness in obesity (ACE-obesity): an overview of the ACE approach, economic methods and cost results. BMC Public Health 9:419.

Crocker DD, Kinyota S, Dumitru GG, Ligon CB, Herman EJ, Ferdinands JM, et al. 2011. Effectiveness of home-based, multi-trigger, multicomponent interventions with an environmental focus for reducing asthma morbidity: a community guide systematic review. Am J Prev Med 41(2 Suppl 1):S5-32.

Daly J, Willis K, Small R, Green J, Welch N, Kealy M, et al. 2007. A hierarchy of evidence for assessing qualitative health research. J Clin Epidemiol 60(1):43-49.

Eden J, Levit L, Berg A, Morton S, eds. 2011. Finding what works in health care: Standards for systematic reviews. Washington, D.C.: The National Academies Press.

Fitzpatrick-Lewis D, Yost J, Ciliska D, Krishnaratne S. 2010. Communication about environmental health risks: a systematic review. Environmental health: a global access science source 9:67.

Glied S, Tilipman N. 2010. Simulation modeling of health care policy. Annu Rev Public Health 31:439-455.

Guyatt GH, Oxman AD, Vist GE, Kunz R, Falck-Ytter Y, Alonso-Coello P, et al. 2008. GRADE: an emerging consensus on rating quality of evidence and strength of recommendations. BMJ 336(7650):924-926.

Kung J, Miller RR, Mackowiak PA. 2012. Failure of Clinical Practice Guidelines to Meet Institute of Medicine Standards: Two More Decades of Little, If Any, Progress. Arch Intern Med: 1-6.

Lu Y, Yao Q, Gu J, Shen C. 2013. Methodological reporting of randomized clinical trials in respiratory research in 2010. Respiratory care. doi:10.4187/respcare.01877.

Merlin T, Weston A, Tooher R. 2009. Extending an evidence hierarchy to include topics other than treatment: revising the Australian 'levels of evidence'. BMC medical research methodology 9:34.

Moher D, Hopewell S, Schulz KF, Montori V, Gotzsche PC, Devereaux PJ, et al. 2010. CONSORT 2010 Explanation and Elaboration: Updated guidelines for reporting parallel group randomised trials. J Clin Epidemiol 63(8):e1-37.

National Health and Medical Research Council. 2013. Climate change. Available: http://www.nhmrc.gov.au/your-health/climate-change [accessed 16 June 2014].

Papoutsakis C, Priftis KN, Drakouli M, Prifti S, Konstantaki E, Chondronikola M, et al. 2013. Childhood overweight/obesity and asthma: is there a link? A systematic review of recent epidemiologic evidence. Journal of the Academy of Nutrition and Dietetics 113(1):77-105.

Petticrew M, Chalabi Z, Jones DR. 2012. To RCT or not to RCT: deciding when 'more evidence is needed' for public health policy and practice. J Epidemiol Community Health 66(5):391-396.

Schulz KF, Altman DG, Moher D. 2010. CONSORT 2010 Statement: Updated guidelines for reporting parallel group randomised trials. J Clin Epidemiol 63(8):834-840.

Schulz C, Moller MF, Seidler D, Schnell MW. 2013. Evaluating an evidence-based curriculum in undergraduate palliative care education: piloting a phase II exploratory trial for a complex intervention. BMC Med Educ 13:1.

Stroup DF, Berlin JA, Morton SC, Olkin I, Williamson GD, Rennie D, et al. 2000. Meta-analysis of observational studies in epidemiology: a proposal for reporting. Meta-analysis Of Observational Studies in Epidemiology (MOOSE) group. JAMA 283(15):2008-2012.

Vandenbroucke JP, von Elm E, Altman DG, Gotzsche PC, Mulrow CD, Pocock SJ, et al. 2007. Strengthening the Reporting of Observational Studies in Epidemiology (STROBE): explanation and elaboration. Epidemiology 18(6):805-835.

von Elm E, Altman DG, Egger M, Pocock SJ, Gotzsche PC, Vandenbroucke JP. 2008. The Strengthening the Reporting of Observational Studies in Epidemiology (STROBE) statement: guidelines for reporting observational studies. J Clin Epidemiol 61(4):344-349.

Wiley KE, Zuo Y, Macartney KK, McIntyre PB. 2013. Sources of pertussis infection in young infants: a review of key evidence informing targeting of the cocoon strategy. Vaccine 31(4):618-625.
